# Relapsing periodic arthritis, palindromic rheumatism and *MEFV* gene-related variants alleles in children

**DOI:** 10.1186/s12969-019-0329-2

**Published:** 2019-06-06

**Authors:** Farhad Salehzadeh, Manuchehr Barak, Roghayeh Nematdoust Haghi

**Affiliations:** Pediatric Department, Bouali Children’s Hospital, Ardabil University of Medical Sciences (ARUMS), No 105 Shahrak Azadi, Azerbaijan Streets, Ardabil, 56157 Iran

**Keywords:** *MEFV* gene, Palindromic rheumatism, Recurrent synovitis of hip, FMF-related arthritis

## Abstract

**Background:**

Relapsing periodic arthritis is a general term used for a group of diseases with recurring and periodic nature, in which the joints are intermittently involved. The aim of this study is to evaluation of the possible relationship between *MEFV* gene mutations in intermittent arthritis of children which has recurring and periodic nature.

**Methods:**

In this cross-sectional study we reviewed medical records of all patients with recurrent and periodic arthritis referred to pediatric rheumatology clinic from 2003 to 2019.We excluded all patients with suspicious anamnesis and/or positive history of FMF, JIA, gout, psoriasis, IBD and SLE. The peripheral blood of these patients was screened for the 12 common pathogenic *MEFV* gene variants (FMF Strip Assay, Vienna lab, Vienna, Austria) according to manufacturer’s instructions.

**Results:**

Among 195 recorded files, 11 patients were identified with idiopathic recurrent and periodic feature. 3 patients suffering from recurrent transient synovitis hip (RSH) and 8 patients were fully compatible with the Pasero and Barbieri modified criteria of palindromic rheumatism (PR). Their mean age at diagnosis was 6.5 and 4.6 years and the average follow-up was 2.6 and 6.6 years in PR and RSH patients, respectively. The most frequently involved joints were knees and ankles in PR patients. The mean duration of attacks was 3.3 days in RSH patients and with various courses in PR. The median number of attacks was 4.75 in PR and 3 in RSH patients per year. In PR group 7 patients showed no mutations and all patients of RSH had these mutations; V726A, R761H, A744S as in heterozygote pattern.

**Conclusion:**

An idiopathic relapsing periodic arthritis in children with exclusively hip involvement is a *MEFV* gene related arthropathy; whereas with various joint attacks it could be considered as childhood PR.

## Background

The term Relapsing periodic arthritis has been proposed to describe a group of arthritis that has recurring and periodic nature, in which the joints are intermittently involved. It may result from hereditary, acquired, or genetic and/or infectious background [1]. These include crystalline arthropathies, such as gout;inflammatory bowel disease (IBD)-related arthritis; Behcet’s syndrome; Whipple’s disease; relapsing polychondritis; intermittent hydrarthrosis and arthropathy of familial Mediterranean fever (FMF) [2].

One of this arthritis is Palindromic Rheumatism (PR), entity originally described by Hench and Rosenberg in 1944; however, is not well-defined accurately in pediatric rheumatic diseases. It is a periodic idiopathic arthritis characterized by sudden, transient, recurring, episodes of mono or oligo-arthritis, is not associated with imaging abnormalities, [3] and lasting from a few hours to several days, which resolved spontaneously without residual joint damage, and is associated with positive acute phase reactant during attacks [4, 5].

The most commonly affected joints are the knees, wrists, shoulders, ankles, and small joints of the hand. The hips, feet and elbows are less often involved; however, PR can affect any joint [6].

It affects adults of both sex [7] and due to the intermittent and self-limited symptoms; diagnosis can be difficult or delayed. There is not distinct test could confirm the diagnosis [2].

The *MEFV* gene is located on the short arm of chromosome 16 at position 13.3(16p 13.3) [8]. *MEFV* gene was predominantly expressed in monocytes and granulocytes, [9] both of which have major roles in the pathophysiology of inflammatory and auto-inflammatory disease at the acute phase and evolution of cytokines storm, an important immunological abnormality in KD [10].

It has been proposed that *MEFV* mutations might increase the baseline of inflammation, induced the development of inflammatory diseases, and affect the clinical course of these disorders [11].

To clarify the correlation and relationship of idiopathic relapsing periodic arthitis (IRPA) of childhood, such as pediatric Palindromic Rheumatism with common pathogenic *MEFV* gene mutaions, this study has been planned.

## Material and methods

### Patients

This is a retrospective, case series, cross sectional study and included the patients with IRPA during 16-years from 2003 to 2019 at pediatric rheumatologic clinic. To identify patients, we reviewed all patients’ files with recurrent arthritis. Patients with suspicious anamnesis and/or positive histories of FMF, JIA, SLE, gout, psoriasis, IBD, celiac and infectious causes such as brucellosis were excluded. None of the patients and their first degree families had FMF and FMF related symptoms. Arthritis had been confirmed by imaging including ultrasound and or MRI further than laboratory tests.

Tel-Hashomer criteria have been used to diagnosis of FMF, and for PR diagnosis, Pasero and Barbieri criteria with some modification was committed (Table 1). Retrieved data were entered into a researcher designed questionnaire, containing demographic, characteristics, imaging different consultations and laboratory findings of patients. Patients had taken treatment in short course such as NSAID and Hydoxychloroquine (HCQ) intermittently as DMARDS with suspicion of unclassified and atypical JIA (CLASS 7) [1]. The study is complaint with the Helsinki Declaration and was approved by the local Ethics Committee under number (IR.ARUMS.REC.1396.95). Informed consent was obtained from all the participants, and/or their parents.

**Table 1:** Diagnostic criteria for palindromic rheumatism

Pasero&Barbieri (1986) [12]	
▪ A history of brief sudden-onset, recurrent attacks of monoarthritis	
▪ Direct observation of one attack by a physician	
▪ Three or more joints involved in different attacks	
▪ More than five attacks in the last 2 years	
▪ Negative x-rays, acute phase reactants and rheumatoid factor	
▪ Exclusion of other recurrent monoarthritis: gout; chondrocalcinosis; intermittent hydrarthrosis; and periodic diseases	

Unlike in the adult population, the differential diagnosis of pediatric periodic arthritis and PR may include a number of clinical entities, such as playground and sport mechanical injuries; Ehlers Danlos and joint hypermobility; post-infectious arthritis, (such as post streptococcal reactive arthritis); pain amplification syndromes and osteochondrosis. These possibilities, as well as other domestic disease such as brucellosis were carefully ruled out by the treating pediatric rheumatologists after extensive workup.

### MEFV gene mutation analysis

Peripheral blood was collected from 11 patients and the samples were screened for the 12 common pathogenic variants(E148Q, P369S, F479 L, I692del, M680I(G/C), M680I(G/A), M694 V, M694I, K695R, V726A, A 744S,R 761H) by an RDB assay (FMF Strip Assay, Vienna lab, Vienna, Austria) according to manufacturer’s instructions, from fresh blood sampling simultaneously at genetic lab center of the hospital.

## Result

Among 195 recorded files, 11 patients matched IRPA and were reviewed thoroughly. First group included 8 patients who their symptoms were compatible with PR, 6 of them were male and second group contained 3 patients suffering from recurrent synovitis of hip (RSH). Demographic characteristics of these patients are presented in Table 2.

**Table 2:** Demographic data of IRPA patients

	Palindromic Rheumatism (PA)	Recurrent synovitis of the hip (RSH)
The mean age (years)	10.12	10.33
Age at diagnosis (years)	6.5	4.6
Male/female (N)	6/2	1/2
Duration of follow-up ^a^ (years)	2.6	6.6
Number of episodes per year	4.75	3
Attack intervals (mean) month	3.1(*n* = 7)	1.6
	Variable(*n* = 1)	
Systemic symptoms during attacks (%)	37.5%	0
Morning stiffness during attacks (%)	12.5%	0
Join involvement	Hip(n = 1)	
	Knee(*n* = 3)	
	Ankle(n = 3)	Hip(n = 3)
	Variable(n = 1)	
Joint pattern	Monoarticular	Monoarticular
Mutations	Non (n = 7)	A744S(Female)
	V726A(n = 1/male)	R761H(Female)
		V726A(Male)
Average duration of episodes	3.5 days(*n* = 4)	
	2.3 h(n = 3)	3.3 days
	Variable(n = 1)	

^a^Defined as the time from first presentation to last follow-up

In the clinical evaluations of patients with PR; hip, knee and ankle joints involvements were observed in 1(12.5%), 3(37.5%) and 3(37.5%) patients, respectively. In one patient, in each episode, different pattern of joints (hip, knee, ankle, and wrist) were involved.

### Laboratory and radiographic findings

In PR, during flares, C-reactive protein (CRP) and the erythrocyte sedimentation rate (ESR) in 5 patients was higher than normal. RF, antinuclear antibodies (ANA), HLAB27, Anti–dsDNA, Anti-ccp, and ANCA were negative in patients. Ophthalmological examination had normal results.

In RSH, during flares, CRP and ESR were near normal. RF and ANA, HLAB27, Anti–dsDNA, Anti-ccp, and ANCA were negative. X-ray was normal, although the Ultrasound showed the synovitis with variable degree of synovial effusion. Radioisotope scans were normal.

## Discussion

This study attempts to assess and characterize idiopathic relapsing and periodic arthritis (IRPA) in pediatric and clarify their possible relationship with *MEFV* gene variant alleles. This study includes 8 patients who their features were compatible with Palindromic Rheumatism and 3 patients with RSH.

Most of PR group were male and the knee and ankle were the most commonly affected joints. FMF is not a rare disease in this area, [13] and FMF related arthritis is not uncommon features; however, among PR group, seven patients had negative results for *MEFV* gene mutations. It was observed only in one patient with PR features (12.5%) as heterozygote V726A mutation. This mutation is a very rare variant of healthy and normal population of this area (2.2%)(our under published data; however, its original language publishing is available at www.arums.ac.ir According to Beheshtian’s report that has been planned from all part of Iran, in northwest and Azeri healthy people the frequency of *MEFV* alleles were 13.2 and 10.3% respectively. From total 9 mutant alleles 7 were E148Q and the rest were P396Q, and V726A, whereas R761H and A744S alleles have not been shown among healthy people [14]. Whereas in our study overall mutations alleles were 25 and 18.3% of them was E148Q.)

This study displayed lower frequency rate of *MEFV* variants alleles in childhood IRPA with PR phenotype.

There is not similar pediatric study in our knowledge; however, in a study by Cañete et al.,in adults [15] 65 patients with Palindromic Rheumatism were evaluated,12.3% of patients with PR had *MEFV* gene mutations (carried at least 1 mutated *MEFV* allele). Kobayashi et al. reported the E148Q allele is the most common encountered allele in patients with PR [16].

Cabrera-Villalba concluded that in a number of patients with Palindromic Rheumatism *MEFV* gene mutations can be observed, but this mutation is not likely to be related to the pathogenesis of the disease [1].

In our study, all 3 patients with RSH had *MEFV* gene mutations that including R761H, V726A, and A744S in heterozygote form which are not variants of our healthy and normal population (our under published data).

Although RSH is not a common condition in children and this study include only three cases, the high rates of *MEFV* mutations in RSH patients emphasis the potential role of *MEFV* gene alleles as a pathogenic background in this group. Peleg suggests the possible role of *MEFV* gene in different rheumatic disease other than FMF [17], accordingly RSH could be considered as a *MEFV* gene related arthritis.

Although the outcome of children with one *MEFV* mutation in the populations, in which FMF carrier frequency is high, continues to be a challenge for physicians, Makay recommend periodic clinical visits, particularly if the child has a pathogenic *MEFV* mutation [18].

It is possible that some of these patients develop FMF related symptoms in the future, during their third and or more decades. If it occurs we will confront with a new dilemma; whether RSH is the first presentation of FMF-related arthropathy, although it has not been reported up to now, and or it is a separate entity and co-existed with FMF.

## Conclusion

In our opinion, an idiopathic relapsing periodic arthritis in children with exclusively hip involvement is a *MEFV* gene related arthropathy; whereas with various joint attacks it could be considered as childhood PR, a condition that has not been affected by *MEFV* gene related inflammation.

Although the number of patients in these results is too low and a large sample size will be required to confirm this finding, however; IRPA is not a common condition in children.

## Data Availability

Please contact first author (Salehzadeh F.) for data request.

## Abbreviations

FMF: Familial Mediterranean Fever
IRPA: Idiopathic relapsing and periodic arthritis
MEFV: Mediterranean Fever Gene
PR: Palindromic Rheumatism
RSH: Recurrent Synovitis of Hip
